# Lung Ultrasound for Pleural Effusion in Cancer Patients: Advanced Ultrasound for Pleural Lesions—A Narrative Review

**DOI:** 10.3390/cancers18010038

**Published:** 2025-12-22

**Authors:** Hajo Findeisen, Christian Görg, Viktoria Zies, Michael Ludwig, Christoph F. Dietrich, Amjad Alhyari, Corinna Trenker-Burchert

**Affiliations:** 1 Department for Hematology, Oncology and Immunology University Hospital Giessen and Marburg, Philipps University Marburg, 35043 Marburg, Germany; 2 Interdisciplinary Center of Ultrasound Diagnostics, Department of Gastroenterology, University Hospital Giessen and Marburg, Philipps University Marburg, 35043 Marburg, Germany; 3 Department for Internal Medicine, Red Cross Hospital Bremen, 28199 Bremen, Germany; 4 Department for Internal Medicine, Hospital of the German Armed Forces, 10115 Berlin, Germany; 5 Department Allgemeine Innere Medizin (DAIM), Kliniken Hirslanden Beau Site, Salem und Permanence, 3013 Bern, Switzerland; 6 Department of Gastroenterology and Hepatology, Asklepios Klinik Weissenfels, 06667 Weissenfels, Germany

**Keywords:** lung cancer, pleural disease, thoracic imaging, lung ultrasound, pleural effusion, CEUS, elastography

## Abstract

**Background:** Pleural effusion (PE) is a frequent complication in patients with malignancies and is often associated with poor prognosis. Lung ultrasound (LUS) has become an indispensable bedside tool for detecting, characterizing, and guiding the management of pleural effusions. **Methods:** This narrative review summarizes the current evidence on the diagnostic performance of LUS for PE in cancer patients, emphasizing recent advances in functional ultrasound techniques. **Results:** B-mode LUS can detect small-volume effusions and estimate their volume. Sonographic features such as echogenicity, septations, and pleural abnormalities can help differentiate transudative from exudative effusions. Shear-wave elastography and contrast-enhanced ultrasound provide additional functional information on tissue stiffness and perfusion. This information may help distinguish between malignant and benign pleural lesions and facilitate targeted biopsy when cytology is nondiagnostic. Compared with computed tomography, LUS offers superior evaluation of juxtadiaphragmatic and pleural surface abnormalities. It facilitates safe, real-time thoracocentesis. Recent innovations, including improved quality, affordable handheld ultrasound systems and artificial intelligence-based analysis, are expected to further enhance diagnostic precision and accessibility. **Conclusions:** Although LUS is a sensitive and versatile tool for assessing PE in cancer patients, it has limited diagnostic accuracy in distinguishing between benign and malignant effusions. Advanced techniques, such as shear-wave elastography and contrast-enhanced ultrasound, may further support the differentiation of malignant and benign diseases. Ongoing technological advances are likely to enhance the diagnostic accuracy and accessibility of lung ultrasound.

## Simple Summary

Lung ultrasound is typically the first-line imaging modality for detecting or excluding pleural effusion. Malignant pleural effusion represents an advanced disease stage, with imaging and pathology playing central roles in diagnosis. This review summarizes recent advances in lung ultrasound, particularly contrast-enhanced ultrasound and elastography, which hold promise for improving the diagnostic workup in malignant pleural effusion.

## 1. Introduction

Over 60 years ago, in 1964, Pell was the first to describe a pleural effusion (PE) by using A-mode ultrasound [1]. Shortly afterwards, its potential for guiding thoracocentesis was demonstrated [2]. Since then, sonographic detection of PE has remained the cornerstone in lung ultrasound (LUS) over the past few decades. B-mode LUS has been used to study malignant pleural effusion (MPE) for over 35 years [3]. However, sensitivity and specificity are limited [4]. Thus, improved diagnostics with newer ultrasound methods, such as contrast-enhanced ultrasound (CEUS) and shear-wave elastography (SWE), were studied over the past three years. Their application in assessing PE and pleural lesions is not included in the current European Federation of Societies for Ultrasound in Medicine and Biology (EFSUMB) guidelines on contrast-enhanced ultrasound (CEUS) and shear-wave elastography (SWE) [5,6], and this review summarizes them.

Pleural effusion is a common condition, with an annual incidence of approximately 1.5 million cases in the United States [7]. Population-based data from comparable studies report an incidence of 0.0032% in Bohemia (Czech Republic) [8] and 0.0047% in China [9]. Several comprehensive reviews and studies covering the etiology and management of PE, beyond the scope of MPE, have already been published [7,10,11,12]. A malignant etiology was identified in 14–52% of cases, depending on the clinical context [9,11,12,13]. Due to multimorbidity, PE has multiple causes in a quarter of cases [14]. Anecdotally, bilateral PEs arising from different etiologies have been described and named Contarini’s condition [15]. Additionally, approximately 15% of patients with metastatic lung, breast, ovarian, or gastrointestinal cancers, as well as hematological malignancies, are known to develop a PE [7,16]. Malignant pleural effusion is seen in up to 90% of cases of pleural mesothelioma [17] and in up to 40% in patients with lung cancer [18]. Regardless of whether the MPE is already present at the initial tumor diagnosis or develops due to tumor progression, it always indicates stage IV tumor disease. Moreover, paramalignant pleurisy with PE due to airway obstruction, atelectasis, pulmonary embolism, pneumonia, or caused by radiation should be considered in cancer patients [7]. In most diseases, the presence of PE signifies active and progressive pathology. Consequently, PE is associated with a high one-year mortality rate in patients with cardiac (50%), renal (46%), and liver (25%) failure [19]. In MPE, the one-year mortality rate is even higher, reaching 77% [20]. Breathlessness is the most common symptom in patients with MPE [21]. However, asymptomatic non-small-cell lung cancer (NSCLC) patients and MPE remain at risk of later symptomatic progression [22].

Despite advances in our understanding of pleural carcinomatosis, such as tumor-induced vascular leakage, inflammation, neovascularization, and reduced lymphatic drainage [23,24], the pathophysiological mechanisms determining which patients develop MPE remain poorly understood. Since even minimal MPE is linked to worse prognosis, both understanding its pathophysiology and ensuring its early detection are of high clinical relevance [25,26].

Therefore, LUS plays a pivotal role in diagnosing or excluding PE, as it is highly sensitive (92–100%) and specific (88–100%) compared to computed tomography of the chest (CT) [27,28]. Using LUS to diagnose PE is not only easy, convenient, independent, radiation-free, cost-effective, and more CO_2_-efficient than cross-sectional imaging, but it also enables the identification of important differential pleural and pulmonary diagnoses such as pneumonia [29], interstitial pattern [30], pulmonary embolism [31,32], pleural plaque, and exclusion of a pneumothorax [33].

## 2. Ultrasound for Malignant Pleural Effusion

### 2.1. Quantification

The first step in quantifying MPE is to confirm its presence. If possible, the patient should be examined in a sitting position to optimize visualization of the costodiaphragmatic recesses. Subsequently, the effusion can be categorized semi-quantitatively as small, medium, large, or massive. Even minimal PE, as small as 5 mL, can be detected using LUS [27]. One study suggested estimating PE volume by counting intercostal spaces [34]. Furthermore, several equations have been proposed to quantify the volume of anechoic PE [35,36,37]. In general practice, the volume of pleural effusion in milliliters is estimated by multiplying the lateral effusion width in centimeters by 90 [36,37].

### 2.2. Echogenicity

Echogenicity should be described in the diagnostic evaluation of PE. Yang et al. categorized pleural effusions as anechoic, homogeneously echoic, complex non-septated, or complex septated [38]. Of 113 cases of MPE, 40.7% were anechoic, 7.1% were homogeneously echoic, 20.3% were complex non-septated, and 31.9% were complex septated. This study demonstrated that echogenicity alone is not specific to MPE; however, exudative effusions are more likely to be present when the PE is non-anechoic. Subsequent investigations reported anechoic MPEs in 4.5–42.4% of cases [39,40,41,42,43]. When a complex septated PE is detected, infectious causes and hemothorax must also be considered [38,41,42,43,44]. Chian et al. described the “swirling sign” as a specific finding of MPE in patients with known malignancy [45]. However, subsequent studies have failed to confirm this observation [44,46,47,48]. The initial findings are likely to be explained by selection bias and a high pre-test probability of MPE in the studied population. Chest CT is also unreliable for distinguishing between transudates and exudates [49]. In contrast, LUS has been shown to be more sensitive than chest CT for detecting complex septated PE (82.6% vs. 59.8%) and is therefore especially useful before and during thoracocentesis [50]. Furthermore, repeated thoracocentesis can lead to pleural inflammation and fibrin strands, resulting in complex septated effusions [51].

#### Additional Ultrasound Techniques

**Figure 1:**
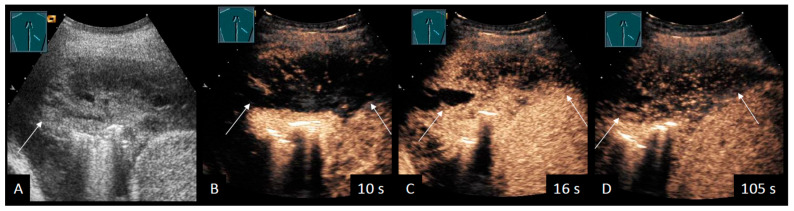
Patient with MPE due to malignant B-cell lymphoma. (**A**) B-mode US showing hyperechoic, complex septated PE (arrow). (**B**) Peripheral systemic arterial supply is demonstrated by CEUS after 10 s. (**C**) Arterial hypoenhancement is inhomogeneous, and non-perfused areas of the PE are revealed. (**D**) A decreasing parenchymal hypoenhancement is registered in comparison to the nearby liver parenchyma after 100 s.

A small-sample study investigated PE using SWE to differentiate transudate and exudate, reporting higher mean SWE velocities in exudative PE [52]. A few case reports have used CEUS to evaluate complex PE, showing enhanced septations in MPE [53,54] (Figure 1).

### 2.3. Pleural Lesions

In LUS imaging for MPE, the detection and assessment of pleural changes play a crucial role. Although the sensitivity of malignant pleural thickening and nodularity is only moderate, specificity is high, indicating a high likelihood of pleural carcinomatosis (see Table 1) [3,4,38,39,42,44,53,55,56,57,58], particularly in patients with a high pre-test probability of MPE. Most studies have used a cut-off of >1 cm to define malignant pleural thickening, since thinner pleural thickening is more commonly observed in benign PE [39,42,44,50,55,56,58]. A meta-analysis found that, compared to other sonographic features, pleural nodules have the highest positive predictive value for diagnosing MPE by LUS, making them strongly indicative of malignant involvement [4,59,60].

#### Additional Ultrasound Techniques

To overcome the limited diagnostic accuracy of B-mode LUS in defining pleural lesions, small studies have investigated the use of additional techniques such as SWE or CEUS. Notably, Jiang et al. primarily investigated pleural thickening with SWE, providing a cut-off of 47.25 kPa with good sensitivity (83.64%) and specificity (90.67%) for distinguishing MPE from benign conditions [61]. This improves the overall diagnostic accuracy compared to B-mode LUS alone (sensitivity 60%) [61]. In another investigation, SWE was applied to the intercostal spaces superficial to the PE in patients presenting with unclear unilateral PE in the emergency department [57]. Using an intercostal space SWE (max) cut-off value of 2.01 m/s, all MPEs were identified; however, specificity was reduced, resulting in a higher false positive rate for benign PE [57]. The pathophysiological basis for increased intercostal stiffness remains unclear, as the authors did not specify the region of interest or its diameter, making it difficult to determine precisely which tissue was assessed by SWE. Overall, elastography of pleural thickening and nodules seems to be a promising addition to B-mode LUS. Nevertheless, SWE cannot reliably distinguish exudative benign PE from MPE, as reported in a conference abstract [62].

**Figure 2:**
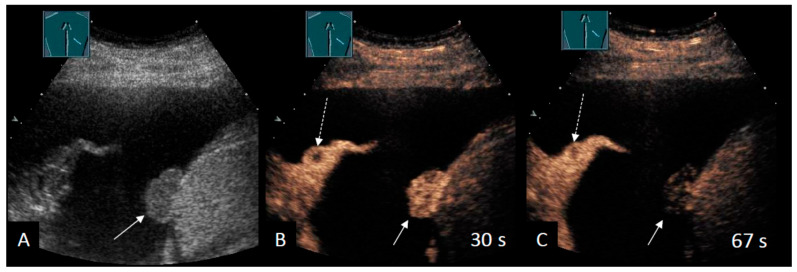
Patient with right-sided MPE secondary to endometrium cancer. (**A**) B-mode US demonstrates an inhomogeneous atelectatic tissue as well as a 2 cm hypoechoic pleural nodule located on the diaphragm (arrow). (**B**) In the arterial phase, CEUS demonstrates marked, inhomogeneous enhancement (arrow), along with a hypoechoic, round intrapulmonary lesion suggestive of an additional lung metastasis (dotted arrow). (**C**) The diaphragmatic lesion exhibits a rapid washout within one minute, in contrast to the persistently enhanced adjacent liver parenchyma (arrow).

Even so, CEUS can be used to differentiate between malignant and benign PE, as illustrated in a pictorial essay [55]. In a retrospective study, 83 patients with PE of unknown cause were evaluated [53]. The authors found that CEUS improved the diagnostic accuracy from 66.7% to 87.5% in patients with cytology-negative PEs suspicious for malignancy [53]. Nevertheless, overall sensitivity and specificity remained moderate. Several studies demonstrated that malignant parietal pleural lesions show marked perfusion [53,56,58], whereas benign lesions show marked (45.8%) or reduced (54.2%) enhancement [56]. In a prospective CEUS study by Yang et al., malignant pleural lesions (predominantly parietal) exhibited a shorter arrival time, a faster time-to-peak, and a higher time-intensity curve than benign pleural disease. This confirms the results quantitatively. The authors proposed a “fast-in/fast-out” enhancement pattern for malignant pleural lesions (Figure 2) and reported sensitivities of 93.3% and specificities of 90.0% for distinguishing pleural lesions in their cohort [58].

### 2.4. Intrapulmonary Lesions

Another important aspect is the use of PE as an acoustic window through which to evaluate the underlying atelectatic lung tissue, as has been demonstrated for over 30 years [63]. To date, only a limited number of LUS studies have reported on the parenchymal or visceral pleural lesions described above [38,42,44,53]. However, these lesions are likewise not specific for distinguishing MPE from benign PE [38,42,44,53].

#### Additional Ultrasound Techniques

**Figure 3:**
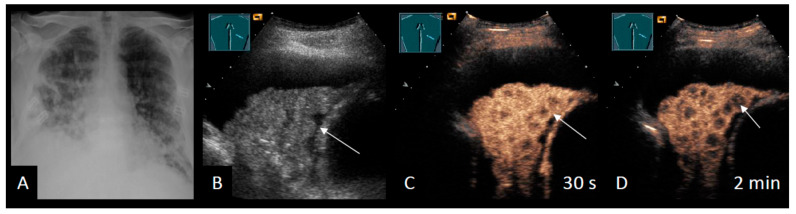
Patient with MPE secondary to renal cell carcinoma. (**A**) A chest X-ray shows multiple, diffuse opacities and a right-sided pleural effusion. (**B**) B-mode US displays an inhomogeneous atelectasis with an irregular surface (arrow). (**C**) CEUS shows an inhomogeneous enhancement with several hypoenhancing intrapulmonary lesions. (**D**) These lesions reveal a marked washout after two minutes, indicating diffuse lung metastases.

To date, only two studies have investigated these pulmonary lesions using elastography, reporting a good diagnostic accuracy [57,64]. However, the accuracy and feasibility of elastographic measurements are limited by motion and depth, and it remains unclear how these measurements are affected by PE. In addition, intraparenchymal malignant lesions have been shown to exhibit significantly more inhomogeneous perfusion than benign lesions [53] (Figure 3). In a subsequent study by the same authors, CEUS improved the detection of tumors within obstructive atelectasis [65]. Furthermore, CEUS has demonstrated substantial clinical relevance when performed prior to transthoracic biopsy of central lung tumors, enabling the differentiation of perfused from non-perfused areas [66].

### 2.5. Comparisons with Chest CT and Positron Emission Tomography–Computed Tomography (PET-CT)

Distinguishing benign PE from MPE remains challenging with B-mode LUS, chest CT, and PET-CT [67,68]. For this reason, several cross-sectional imaging studies have incorporated extrapulmonary findings, such as extrapulmonary metastases or cardiomegaly, into their diagnostic scores [69,70]. In contrast, this approach has so far rarely been applied in LUS studies [39]. This highlights an important strength of cross-sectional imaging, since suggestive findings must either be actively sought sonographically or may remain undetected due to the inherent limitations of LUS. Both imaging modalities are likely complementary. A small prospective investigation recently demonstrated that, as expected, chest CT was more sensitive in detecting mediastinal pathologies, whereas B-mode LUS performed better in assessing juxta-diaphragmatic abnormalities [60]. Furthermore, ultrasound has demonstrated greater sensitivity than CT for detecting cervical lymph node metastases in lung cancer patients [71,72].

Both clinical practice and the current guidelines recommend using LUS early in the diagnostic workup of PE, particularly to guide the mandatory diagnostic thoracocentesis [7,73,74,75]. However, the management of MPE appears to be more influenced by clinical experience, local expertise, and institutional tradition than by robust evidence. Only one study has questioned the additional diagnostic value of B-mode LUS in cases of suspected MPE where a chest CT scan has already been performed [76]. Evidence regarding the use of PET-CT is also conflicting [68,70,77,78]. One guideline states that a PET-CT scan may be considered in cases of suspected MPE when previous histological results were negative or when tissue sampling is not feasible due to anatomical restrictions [79]. However, a recent randomized trial demonstrated that PET-CT-guided biopsy was no more effective than CT-guided biopsy for suspected malignant pleural lesions (90% of which were malignant pleural mesothelioma) [80]. Therefore, expert commentary suggests restricting PET-CT to selected cases [75].

### 2.6. Cytology/Histology

Nevertheless, cytology remains the primary tool for diagnosing MPE, with an overall sensitivity ranging from 46% to 67%, depending on the underlying malignancy [21,81,82]. In a large prospective trial, the sensitivity of cytology was particularly low for diagnosing mesothelioma (6%), whereas it reached 95% for pleural involvement from ovarian carcinoma [81].

**Figure 4:**
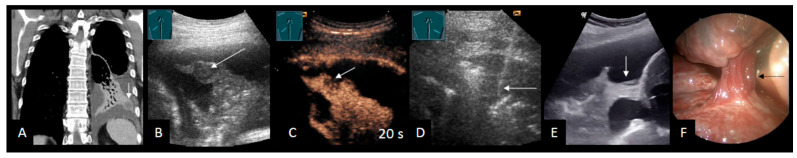
Patient with MPE due to malignant mesothelioma. (**A**) Chest CT showing a left-sided pleural effusion with compression atelectasis, hypodense intrapulmonary nodule (arrow), and an irregular pleural thickening. (**B**) B-mode LUS shows a hypoechoic intrapulmonary lesion (arrow). (**C**) CEUS shows a bronchial arterial, inhomogeneous hypoenhancement of the lung lesion after 20 s. There is also an enhancement of the pleural nodular thickening. (**D**) Following thoracocentesis, transthoracic sonography-guided biopsy was performed (arrow), confirming epithelioid-type malignant pleural mesothelioma in both the parietal pleura and the lung lesion. (**E**) Patient with a right-sided pleural effusion and a pleural adhesion (arrow) between the atelectatic lung and the diaphragm. (**F**) Corresponding thoracoscopic image obtained during assessment for pleural carcinomatosis.

The challenge of the clinical task lies in stratifying the risk of MPE and its underlying malignancy. This allows for the selection of the most appropriate initial diagnostic procedure, reducing the need for repetitive invasive punctures and avoiding delays in diagnosis. For example, asbestos-exposed men with exudative PE have an approximately 60% risk of MPE, yet cytology provides inadequate sensitivity in this population. Consequently, some authors advocate early medical thoracoscopy in such high-risk cohorts [83,84]. Imaging plays an important role in this context, as false-negative cytology is more frequent in MPE with pleural thickening (e.g., mesothelioma) [83,85]. In these cases, real-time US-guided pleural biopsy or thoracoscopy should be considered [86,87,88] (Figure 4).

#### Additional Ultrasound Techniques

A prospective study evaluated the use of elastography-guided biopsy in 98 patients with unclear cytology-negative PE and pleural thickening of up to 5 mm [89]. The procedure achieved an overall sensitivity of 92.9% and a specificity of 88.7% for MPE. Direct comparisons with other studies are limited, as previous reports often included patients with greater pleural thickening and demonstrated lower sensitivity. This may be due to an MPE diagnosis based on cytology [39,44,56,58,87]. This study also experienced difficulty in differentiating fibrinous pleural processes, such as tuberculosis, based on the SWE threshold, resulting in moderate sensitivity. Importantly, no control group was included, so these results require confirmation in other patient populations. Similarly, a prospective single-arm study assessing pleural lesions with CEUS prior to ultrasound-guided biopsy reported a diagnostic accuracy of approximately 99% when CEUS was used to guide sampling [90]. Moreover, CEUS may provide additional diagnostic benefits in the workup of cytology-negative PE [53] (Table 2).

### 2.7. Non-Expandable Lung

As MPE signifies an advanced stage of disease, treatment is primarily focused on controlling symptoms and providing palliative care. Only a small subset of MPE patients, such as those with non-Hodgkin lymphoma, may respond to systemic anti-cancer therapy [91]. The main interventions for symptomatic MPE patients are repeated thoracocentesis, an indwelling pleural catheter, or pleurodesis [7,16,24].

**Figure 5:**
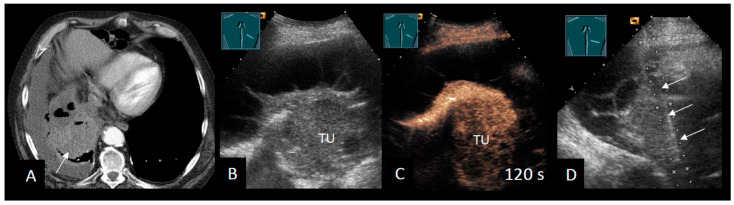
Non-expandable lung in a right-sided MPE secondary to NSCLC. (**A**) Chest CT scan showing a right-sided pleural effusion with lung consolidation (arrow). (**B**) B-mode LUS shows a complex septated pleural effusion and a slightly hypoechoic pulmonary consolidation with loss of the bronchovascular architecture (TU). The lung is further entrapped by fibrous septations. (**C**) CEUS demonstrates an inhomogeneous hypoenhancement at two minutes, which allows clearer delineation of the tumor tissue from the adjacent atelectatic lung parenchyma and excludes non-perfused (necrotic) areas. (**D**) US-guided biopsy is performed (arrows), confirming the diagnosis of NSCLC.

Non-expandable lung (NEL) is a clinically relevant condition that occurs in approximately 33% of MPE cases [92]. It arises from pleural inflammation and fibrosis, as well as tumor infiltration and adhesions, which prevent the lung from fully re-expanding during pleural drainage. Consequently, the lung becomes trapped, leading to cough, dyspnea, and chest pain during thoracocentesis. In some cases, hydropneumothorax may develop after a puncture. Therefore, predicting NEL prior to intervention is important, as it may lead to a change in treatment, as indwelling pleural catheters are currently the preferred therapy option. In recent years, LUS has gained increasing importance alongside clinical assessment and pleural manometry [93]. In routine practice, pleural effusion, as well as the mobility and ventilation of atelectatic lung tissue, are subjectively evaluated (Figure 5). A pivotal study by Salamonsen et al. in 2014 was the first to use speckle tracking to quantify the mobility of the consolidated lung tissue, thereby enabling the prediction of NEL prior to intervention [94]. Building on the sinusoid sign reflecting atelectasis mobility in M-mode [95], the authors demonstrated that reduced mobility is characteristic of NEL [94]. Similar findings have since been reported by other groups [96]. However, the available evidence remains limited due to small sample studies and the predictive value of M-mode for NEL is still modest, underscoring the need for further research.

#### Additional Ultrasound Techniques

To date, only one single study has examined the use of elastography for NEL, evaluating both the intercostal thoracic wall and the consolidated, atelectatic lung using SWE [96]. This approach did not demonstrate any diagnostic benefit, and elastographic techniques currently have no established value in this setting. Similarly, CEUS has not been investigated in the context of NEL and has no current clinical application in this setting.

**Table 2:** Overview of studies investigating pleural lesions and MPE with elastography or CEUS.

Author	Year	Population, Study Design	Objective	Main Finding
Elastography				
Hou et al. [62]	2016	n = 61, design not given	To investigate benign and malignant pleural disease with LUS and SWE	Cut-off: 48.7 kPa-mean Sensitivity: 80.8% Specificity: 80%
Jiang et al. [61]	2019	n = 244, single-center, prospective study with development and validation set	To investigate the efficacy of SWE for diagnosing MPE	Cut-off: 47.2 kPa-mean Sensitivity: 83.6% Specificity: 90.7%
Ozgokce et al. [52]	2019	n = 60, single-center, prospective observational study	To differentiate transudative and exudative pleural effusion with acoustic radiation force impulse	Cut-off value: 2.52 m/s Sensitivity: 91% Specificity: 76.5%
Deng et al. [89]	2023	n = 98, multicentric, prospective study	To investigate the diagnostic accuracy of SWE-guided pleural biopsy	Sensitivity for MPE: 88.7% Sensitivity for tuberculosis: 69.6%
Petersen et al. [96]	2024	n = 49, single-center, prospective observational study	To investigate NEL pre-thoracocentesis with measurements of lung and diaphragm movement and SWE of the pleura	AUC M-mode lung: 81% AUC SWE visceral pl: 59% AUC SWE parietal pl: 57%
Nielsen et al. [57]	2025	n = 39, single-center, prospective observational study	To evaluate shear-wave elastography’s accuracy in detecting malignant pleural effusions in the ED	Sensitivity B-mode: 28.6% Specificity B-mode: 90.6% Sensitivity intercostal SWE: 100% Specificity intercostal SWE: 59.1%
CEUS				
Safai Zadeh et al. [53]	2021	n = 83, single-center, retrospective study	To evaluate the value of CEUS in differentiating malignant from benign PE	Sensitivity B-mode: 69.1% Specificity B-mode: 58.5% Sensitivity CEUS: 73.8% Specificity CEUS: 70.7% Subgroup (cytological negative, high risk for MPE): Sensitivity CEUS: 92.3% Specificity CEUS: 90.0%
Findeisen et al. [56]	2022	n = 63, single-center, retrospective study	To describe the value of CEUS for the differentiation of malignant from benign parietal pleural lesions (Pleural effusion in 50.8%)	Sensitivity CEUS: 92% Specificity CEUS: 54%
Yang et al. [58]	2022	n = 50, single-center, prospective study	To investigate the diagnostic capabilities of B-mode LUS and CEUS in terms of differentiating between benign and malignant pleural diseases	Multivariate logistic regression: Sensitivity 93.3% Specificity 90.0%

## 3. Outlook

The two most recent innovations are handheld devices and artificial intelligence (AI). Technological advances have made handheld devices cost-effective and portable diagnostic tools, enabling physicians to detect PE outside traditional medical facilities [97,98]. This portability facilitates flexibility in patient care and may also contribute to the development of healthcare delivery structures that reduce hospitalizations. Home-based palliative care can benefit from the use of portable ultrasound machines [99]. Artificial intelligence is already transforming the diagnostic capability of LUS [100,101,102]; however, MPE has not yet been investigated in published trials. In the future, pleural segmentation combined with deep learning may enable automated PE quantification [100]. Integrating multiparametric US, including B-mode US, SWE, and CEUS, with AI has the potential to enhance diagnostic performance. Real-time AI-assisted US-guided biopsy may further improve diagnostic accuracy.

## 4. Conclusions

Overall, LUS presents a useful tool for the initial assessment of PE of unknown etiology and for managing (suspected) MPE. Depending on the context, B-mode LUS alone has moderate-to-high specificity for differentiating benign PE from MPE but limited sensitivity. CEUS and SWE can improve diagnostic performance; however, the evidence currently supporting their use is limited and their role in clinical management has yet to be fully established. However, LUS should always be interpreted alongside the clinical context, biochemical parameters, cytology or histology, and complementary cross-sectional imaging studies. Chest CT is used for further comprehensive anatomical staging, while PET-CT is reserved for problem-solving in selected cases where the findings are inconclusive and pleural malignancy is suspected. Despite the promising results of CEUS and SWE, the diagnostic accuracy of LUS remains operator-dependent, and undoubtedly, regional and center-specific diagnostic capabilities vary considerably. Standardized protocols and training are essential to ensure reproducibility, particularly as handheld and AI-assisted systems gain clinical relevance. Further research should focus on direct head-to-head studies comparing SWE/CEUS with medical thoracoscopy in diverse populations, particularly those with suspected MPE and negative cytology results.

## Figures and Tables

**Table 1:** Studies of LUS measuring pleural thickness and proposing cut-off values for the detection of malignant pleural lesions suggestive of malignant pleural effusion.

Study	Pleural Thickness	Diagnostic Yield
Qureshi, 2009 [39]	>10 mm	sensitivity 42%, specificity 95%
Bugalho, 2014 [44]	>10 mm	sensitivity 74%, specificity 86%
Findeisen, 2022 [56]	>15 mm	sensitivity 78%, specificity 74%
Yang, 2022 [58]	>7 mm	sensitivity 63%, specificity 90%

## Data Availability

Data sharing is not applicable to this article as no new data were created or analyzed in this study.

## Abbreviations

The following abbreviations are used in this manuscript:

AI	Artificial intelligence
CEUS	Contrast-enhanced ultrasound
CT	Computed tomography
LUS	Lung ultrasound
MPE	Malignant pleural effusion
NEL	Non-expandable lung
NSCLC	Non-small-cell lung cancer
PE	Pleural effusion
PET	Positron emission tomography
US	Ultrasound
SWE	Shear-wave elastography
